# A clear climatic signal of precipitation and temperature in growth of Scots pine young trees in habitats homogenous in relief, soil and access to light

**DOI:** 10.1186/s40529-025-00472-0

**Published:** 2025-08-07

**Authors:** Ruslan Ianbaev, Svetlana Bakhtina, Yulay Yanbaev, Aleksey Kulagin, Nina Redkina, Vadim Tagirov

**Affiliations:** 1 Scientific and Educational Center, Federal State Budgetary Educational Institution of Higher Education “Bashkir State Agrarian University”, Ufa, Russia; 2 Laboratory of Forestry, Ufa Institute of Biology of Ufa Federal Research Center of Russian Academy of Sciences, Ufa, Russia; 3 Department of Ecology and Life Safety, Federal State Budgetary Educational Institution of Higher Education “Ufa University of Science and Technology”, Ufa, Russia; 4 Institute of Physics and Technology, Federal State Budgetary Educational Institution of Higher Education “Ufa University of Science and Technology”, Ufa, Russia

**Keywords:** Climate, Linear increment, *Pinussylvestris*, Young trees

## Abstract

**Background:**

The purpose of our study was to arrange the monthly temperature and precipitation in order of importance for the growth of Scots pine young trees. Model 25-year-old 405 individuals were selected in 27 forested former agricultural lands. Annual tree height increment in 2009–2013, a period with contrasting amounts of summer precipitation, was measured.

**Results:**

In all the trial plots, we found statistically significant synchronous dynamics between annual growth and monthly precipitation of the previous October and current March. Precipitation in August (significant correlation was in 96.3% of habitats), May (92.6%), July (81.5%), previous November (77.7%), and current June (74.1%) was also important for the increments. The temperature of April and September had a critical importance for plants in all habitats. Previous November and December (the correlation was significant at 81.5% of samples), March (77.8%) were also very important. Temperatures of all other months had a more site-specific effect on the growth of trees or did not affect it.

**Conclusions:**

The results obtained made it possible to quantify monthly climate signals in the *Pinus sylvestris* growth and may be useful in predicting the impact of climate change on the species.

## Introduction

Climate change has a growing but territorially uneven impact on the most important environmental factors, such as local temperature and precipitation (Leskinen et al. 2020). As a result, changes occur in the selective pressure that has shaped the adaptive variation of tree species populations to the local environment over multiple generations (Capblancq et al. 2020). Tree species with their low dispersal capacities and long generation cycles are not able to migrate to more favorable conditions in time (Koralewski et al. 2015), because large-scale forest die-off events have already happened (Lombardo 2017). Forests play a crucial role in mitigating climate change, yet they themselves are subject to considerable pressure resulting from these changes(Golub et al. 2022). Given that tree species differ in their life-history traits and adaptive strategies, the assessment of forest ecosystems requires consideration of species-specific responses to climate change (Pecchi et al. 2019; Hamrick 2004).

Boreal coniferous forests comprise 73% of the world’s *biome* (Machacova et al. 2016) and are of great importance for mitigating the effects of climate change (Pan et al. 2011). This is particularly relevant for Russia, where conifer-dominated stands occupy 538.56 out of 794.51 million hectares (i.e., 67.8%) of recorded forested land (Leskinen et al. 2020). In the country, boreal forests absorb 485.8–535.1 Mt C/year in biomass (Filipchuk et al. 2020). Unfortunately, this group of tree species may prove to be particularly vulnerable to global climate change, especially along the southern boundary of the boreal zone (Venäläinen et al. 2020). For example, the shift of the latitudinal and altitudinal margins of the coniferous species distribution ranges was observed (Shiyatov and Mazepa 2007). Such events may be expected in Russia on a larger scale because the country has the main areas of primary boreal forests of the world (Leskinen et al. 2020) and climate change is more pronounced in the country than its speed in other parts of the world. The average annual temperature anomalies are much higher here than the global values of this parameter (by 2.5 times), especially in summer and autumn. Annual precipitation has been increasing since 1980, with a linear trend since the 70 s of the last century (Leskinen et al. 2020).

Scots pine (*Pinus sylvestris* L.) is the most widely distributed species within its genus and plays a key role in boreal forest ecosystems (Leskinen et al. 2020). Although it constitutes only 15.5% of the coniferous forest area in the Russian part of this biome, it is considered a promising species for studying climate signals. This is due to its high ecological plasticity, presence across a broad geographic range (Pyhäjärvi et al. 2020; Dauylbayev et al. 2024), pronounced sensitivity to changes in temperature and precipitation (Liu et al. 2022), and its occurrence in the southern boreal zone—an area particularly affected by shifts in the distribution of coniferous species under current global warming conditions (Venäläinen et al. 2020).

Results of contemporary research confirm this statement. Region-specific differences, which were associated with summer precipitation at the origin of the species locations, were observed (Seidel et al. 2016). Growth depression was shown in forest-steppe Scots pine stands of the Eastern European Plain (Matveev 2014; OsPanov et al. 2018). Displacement of the pine by more drought-resistant species due to higher drought-induced mortality of the Scots pine was revealed (Aguadé et al. 2015). It is predicted that a part of today's Scots pine climatypes will disappear in Siberia as a result of climate change. Some of them will require moving 700–1200 km to the places of future climatic optima (Tchebakova et al. 2006; Abisheva et al. 2022; Safonov 2022).

Assessing tree height is a prevalent method for understanding tree growth patterns in response to climatic changes, particularly in boreal regions where seasonal shifts are more pronounced (Zuidema and van der Sleen 2022). In this context, “growth” is characterized as the annual increase in height, typically measured by evaluating yearly increments. While the analysis of tree-ring widths is a traditional technique, it can present challenges when applied to younger trees (Hillam 1998). Conversely, measuring the annual height growth of young conifers is a more straightforward process, as it involves calculating the distance between the whorls of lateral branches that emerge each year (Pyörälä et al. 2018; Kamaljanova and Burakanova 2020; OsPanov et al. 2020). Despite this simplicity, the climate-driven growth patterns of coniferous species have not been extensively investigated.

The growth of trees is shaped by a complex interplay of biotic and abiotic elements, such as ecological site characteristics, variations in climate, and competition among species, both within and across different species (Biging and Dobbertin 1995; Fritts 1976; Lévesque et al. 2016; Oberhuber and Kofler 2000; Bourakba et al. 2024). The extent of these influences can differ greatly based on the specific environmental conditions, often complicating the ability to pinpoint the direct impacts of climate. Therefore, to more accurately isolate and evaluate the influence of climatic variables, it is beneficial to conduct studies in settings where the effects of other confounding factors are minimized. Ideal conditions would include uniformity in terrain, climate, soil properties, and light availability. While such homogeneous environments are rarely found within established forest stands, a unique opportunity has arisen in Russia due to the emergence of “new forests” on abandoned former agricultural lands. This phenomenon is a consequence of widespread cropland abandonment following the dissolution of the Soviet Union. Almost a third (31 of 87 Mha hectares) of such lands are in Ukraine, Belarus, and, mainly, in the European part of Russia (Schierhorn et al. 2013; Safonov et al. 2021).

## Research objectives and hypotheses

The aim is to investigate the climate-driven growth patterns of young Scots pine trees established on abandoned agricultural lands in Russia, where the effects of topography, soil variability, light availability, and other confounding factors are largely reduced. By assessing the relationship between climatic variables and annual height increments, this research aims to identify the months during which temperature and precipitation most strongly influence Scots pine growth. The findings will enhance our understanding of how this species responds to climate variability and contribute to the development of informed strategies for sustainable forest management in the context of ongoing climate change.

The objectives of this study are as follows:

1. To analyze the relationship between climatic factors—specifically temperature and precipitation—and the annual height growth of young Scots pine (*P.* *sylvestris*) on various types of non-forested lands, including abandoned agricultural fields, former pastures, and grasslands.
2. To identify the most significant months for temperature and precipitation in determining tree growth.
3. To provide insights into the response of Scots pine to climate change and to inform strategies for sustainable forest management.

By achieving these goals, this research will contribute to a better understanding of the impact of climate change on boreal forests and will inform efforts to mitigate its effects.

The hypotheses of the study regarding the impact of climate on the growth of *P.* *sylvestris* include:

1. There is a positive correlation between precipitation and the annual growth of Scots pine (*P.* *sylvestris*). Higher precipitation levels contribute to increased tree growth. This effect is particularly evident on abandoned agricultural lands, where conditions are more favorable for pine growth compared to grasslands and pastures.
2. Temperature has a negative correlation with the annual growth of Scots pine. Elevated temperatures, especially during the summer, reduce tree growth. This effect is particularly pronounced in grasslands and pastures, where the impact of high temperatures may be more significant than on abandoned agricultural lands.
3. The influence of temperature and precipitation on the growth of Scots pine varies depending on the season. Thus, in the summer months, precipitation plays a decisive role in stimulating growth, while the temperature in the winter and early spring periods has a minor effect. The most favorable for growth are spring and autumn, especially on lands that were previously arable.
4. The growth of pine depends on how temperature and precipitation interact with each other. The influence of each of these factors varies depending on the level of the other. For example, in the summer, too high temperatures can reduce the benefits that the plant receives from rain. This interaction manifests itself differently in different ecosystems—abandoned fields, meadows or pastures. In particular, pine on former agricultural areas usually reacts less to temperature fluctuations compared to other places of growth.

In summary, this research has the potential to yield important insights into how Scots pine responds to climate change and to guide sustainable forest management practices in Russia. Encouraging the growth of Scots pine on previously abandoned agricultural lands could also aid in biodiversity conservation and provide economic advantages in the Uchali region.

## Material and methods

**Fig. 1:**
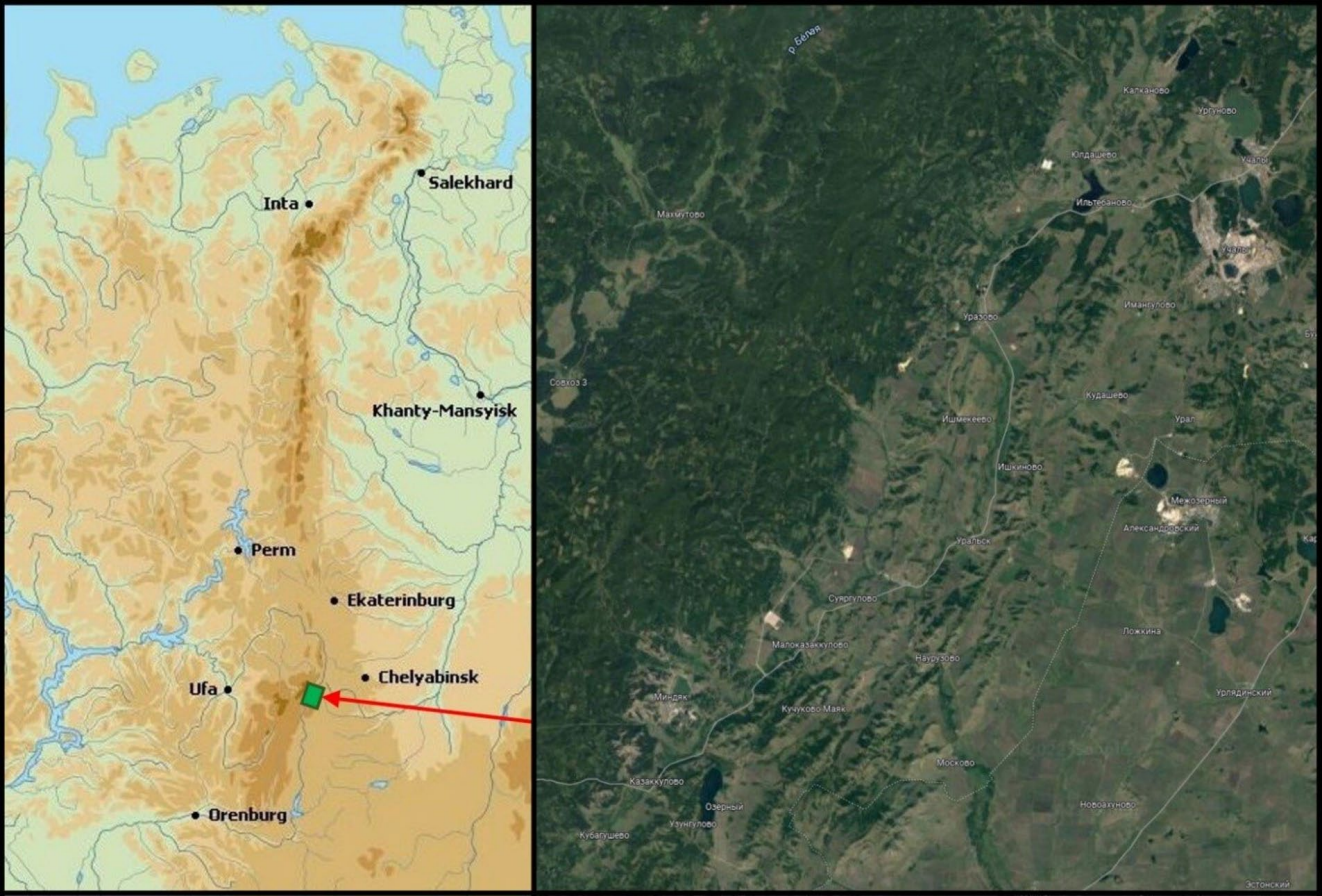
The study area on the Urals map (Source: http://surl.li/acqqmc)

**Fig. 2:**
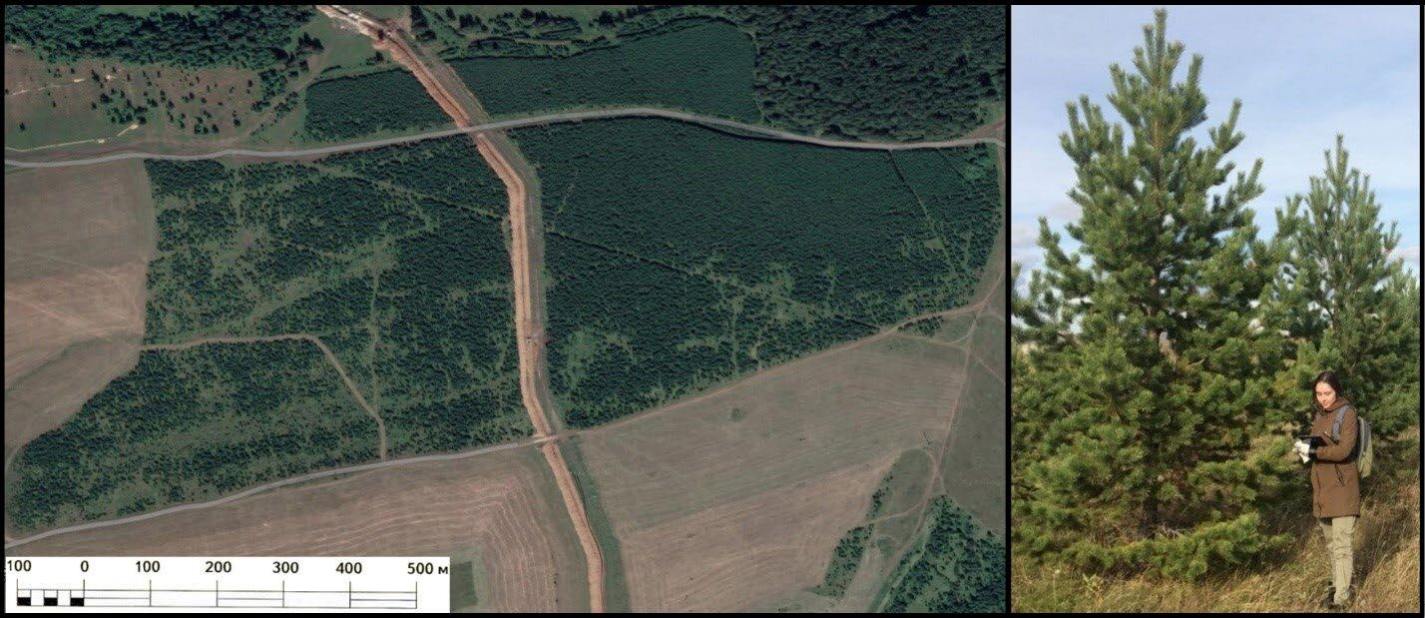
An example of the Scots pine regeneration on abandoned arable lands in the Urals (on the left) and conditions of the tree growth in these habitats (on the right) (Source: Photos taken by the authors and with the assistance of http://surl.li/pixzjn)

The research site is positioned on the eastern slopes of the Southern Ural Mountains, adjacent to the Ural River, which serves as the natural divide between Europe and Asia (see Fig. 1). This region is oriented towards Siberia and the relatively arid areas of Kazakhstan, resulting in a markedly continental climate dominated by anticyclonic conditions. Over the last 134 years, the average annual air temperature in this area has risen from + 3.2 to + 5.7 °C by 2020, while annual precipitation has declined to 353 mm (Nesterenko et al. 2021). The area studied is situated along an 80 km “south-north” transect on the Southern Ural’s eastern macroslope. Scots pine and silver birch (*Betula pendula* Roth.) dominate in the local Uchaly district on 170,716 hectares of forested lands (89.3% of all the stands). Due to this and the properties of pioneer species (Oikonomakis and Ganatsas 2020), these forest tree species have occupied a relatively small part of the local abandoned agricultural territories (Fig. 2) during the last three decades. Ecologically homogeneous plots were selected in these three different types of habitats: 19 former arable lands, 5 grasslands, and 3 pastures. A habitat is a natural environment or type of ecosystem where organisms, such as trees, reside or grow. In the context of this research, the habitats include abandoned lands, pastures, and grasslands, where young Scots pines are found to grow. A plot refers to a specific area of land selected for conducting field research. In this study, 27 experimental plots were chosen, where the annual growth of trees was measured. Each plot is a part of a particular habitat.

We investigated different habitat types within a single region to assess their impact on the growth of young Scots pines, as significant differences in habitat characteristics can exist even within uniform climatic conditions. The consistent factor in this study is the Uchali region, within which the research was conducted, while the habitats differ: abandoned lands, pastures, and grasslands.

In each of the 27 trial plots established on flat areas, 15- to 25-year-old model individuals were selected. In each of the 27 experimental plots, corresponding to different types of former land use, 12 to 15 trees were measured to assess the height growth of young Scots pines. The annual height increment of young Scots pine trees (*P.* *sylvestris* L.) was determined by counting the annual whorls—rings of lateral branches that form around the trunk each year. The distance between adjacent whorls corresponds to the growth for the respective year. Measurements were conducted using a measuring tape with an accuracy of up to 1 cm. To eliminate the influence of light competition, measurements were taken only from isolated individuals located at a minimum distance of 5 m from other trees (Table 1).

**Table 1:** Schematic explanation of the method for measuring annual height increment in Scots pine

Element	Description
1. Age group of trees	Models of 15–25-year-old Scots pine trees were selected
2. Selection of trees for measurement	Twelve to fifteen trees were sampled at each of the 27 plots. Trees were selected at a distance of at least 5 m from one another to eliminate competition effects
3. Determination of tree age	Tree age was determined by counting the number of whorls—annual rings of lateral branches on the trunk
4. Determination of annual increment	The distance between adjacent whorls was measured along the trunk from the base upward
5. Measurement instrument	A measuring tape with an accuracy of 1 cm was used
6. Unit of measurement	Growth was recorded in centimeters as the distance between whorls for each year

The age of the trees was determined by counting the number of annual increments between the whorls, which are formed every year as radial sets of lateral branches around the stem (Pyörälä et al. 2018). To avoid the effect of competition for light access, height measurements were taken only on trees that were located at least 5 m away from neighboring individuals. Annual increments were recorded to the nearest centimeter using a tape measure. Monthly averages of precipitation and air temperature from 2009 to 2013 for the local Uchaly station were obtained from the regional Bashkir Department of Hydrometeorology and Environmental Monitoring. This specific study period was selected for two main reasons. Firstly, it encompasses the most significant variations in summer precipitation over the last two decades, characterized by alternating dry summers and years with exceptionally high rainfall.

Unfortunately, data from other decades were either unavailable or incomplete, which limited the ability to utilize a full dataset. The selection of this specific period was based on its representativeness for identifying particular trends in tree growth under varying climatic conditions. Secondly, at that age, plants were unable to compete with one another for sunlight due to the small size of their crowns not yet closing. Therefore, we aimed to create conditions that would allow us to assess the maximum climate-driven growth response of young trees by minimizing the influence of non-climatic factors that also affect annual plant growth. The meteorological data, which included monthly temperature and precipitation values from 2009 to 2013, were utilized to examine the relationships between variations in climatic factors and the annual growth increments of young trees. To analyze the correlation between annual growth increments and the studied climatic factors, we considered individual months from the previous August leading up to the current September, calculating both seasonal and annual values.

Program STATISTICA 13.3 was used for the descriptive and correlation analyses. After preliminary testing of the data, Spearman’s correlation coefficient R was selected to compare the values of climatic parameters and trees’ annual growth.

To assess the influence of climatic conditions on the annual height increment of trees under conditions of limited climate data, we employed a simplified drought index (DI), which provides an approximate estimate of the balance between moisture availability and thermal load during the critical period of tree vegetation.

The DI index was calculated using the following formula:

$${\text{DI}} = {\text{P}}/{\text{T}}$$

where P is the total atmospheric precipitation (mm) during the summer months (June, July, August); T is the average air temperature (°C) over the same period.

Given the limited sample size for each individual month (n = 5) and, consequently, the low statistical power of the analysis, the Spearman correlation coefficient was applied, as it is less sensitive to sample size and data distribution. This approach is exploratory in nature, aimed at identifying potential trends rather than drawing definitive conclusions.

## Results

**Fig. 3:**
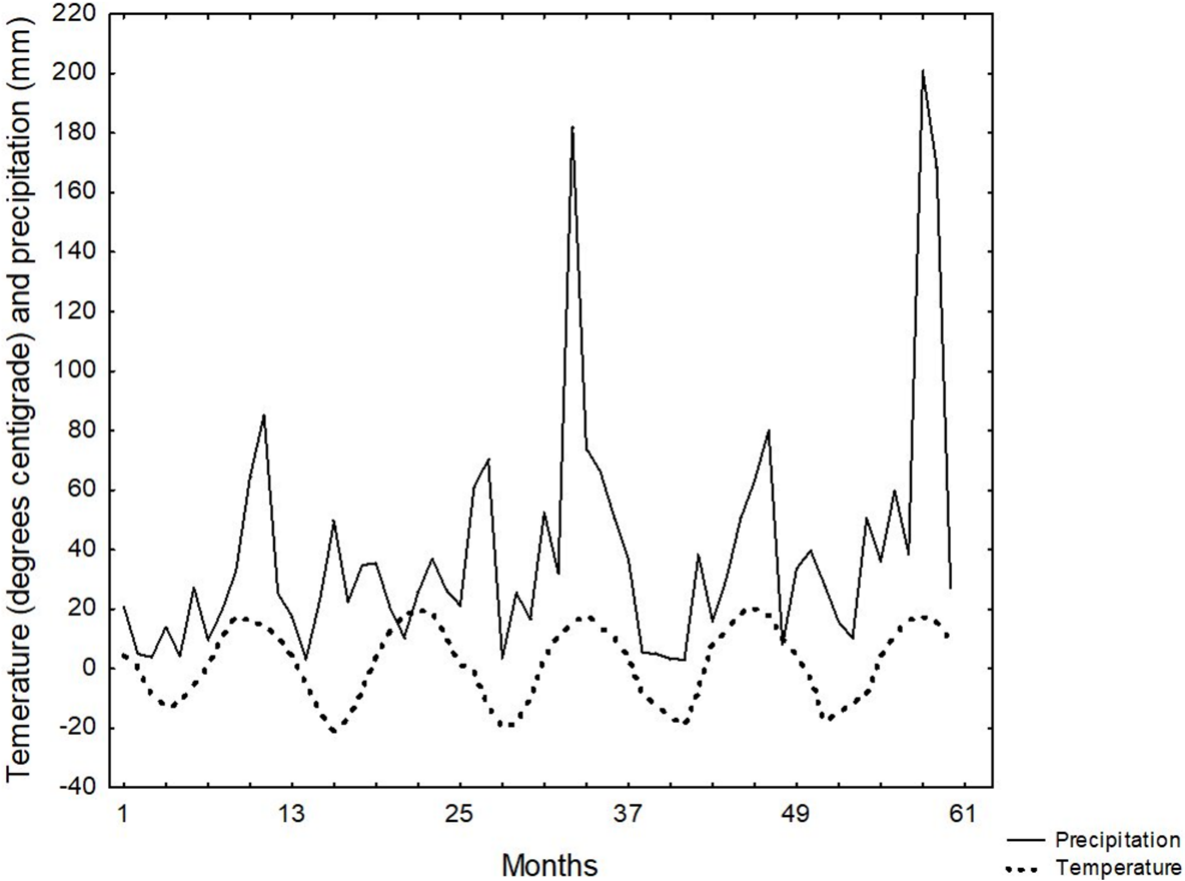
Dynamics of precipitation and temperature from October 2009 to September 2013. *1—October, 2009, 13—October, 2010, 25—October, 2011, 37—October, 2012, 61—October, 2013

During the 60-month study period, temperature changes followed a consistent pattern (Fig. 3) without any extreme values. The bulk of precipitation was concentrated in the summer months, with the notable exception of 2010, which saw only 73.2 mm of rainfall during that period. Two summers, specifically 2011 and 2013, recorded particularly high levels of precipitation. A statistically significant correlation was observed between these climatic factors, with a Spearman correlation coefficient of R = 0.44 (p < 0.001). From 2009 to 2013, snowfall accounted for only 17.6%, 43.0%, 26.4%, 19.5%, and 21.1% of the total annual precipitation, which contributed to the overall trend of reduced precipitation levels during the winter months.

**Fig. 4:**
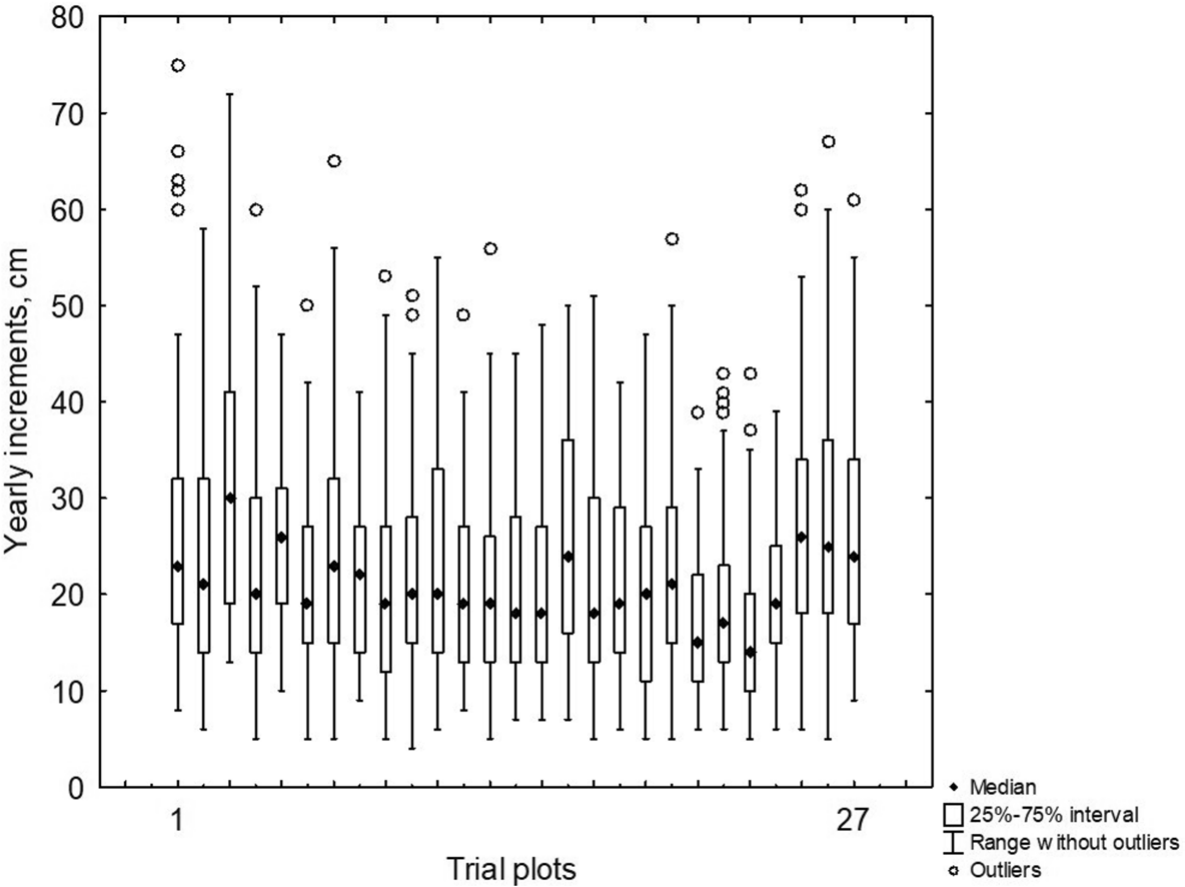
Variation of Scots pine growth in height (2009–2013) within habitats. The ranking is based on the statistical significance of correlation coefficients, with sites demonstrating the strongest correlations ranked highest. Sites occupying the top positions are those where the correlation between precipitation and tree growth is most significant, while those at the lower positions exhibit weaker correlations

Across 27 trial plots, the average yearly growth measurements were as follows: 16.1 ± 0.7 mm in 2009 (ranging from 11.2 to 24.2 mm), 15.4 ± 0.5 mm in 2010 (10.8 to 21.2 mm), 19.6 ± 0.8 mm in 2011 (11.7 to 26.5 mm), 28.5 ± 0.8 mm in 2012 (20.7 to 38.4 mm), and 34.4 ± 1.3 mm in 2013 (22.6 to 51.9 mm). Although the ecological conditions across the various habitats remained largely unchanged, we observed differences in the annual growth of trees among different groups (as shown in Fig. 4). These differences were consistently apparent when we analyzed tree growth over individual years. Our study found that young trees located on abandoned agricultural land demonstrated a higher growth rate, averaging 24.3 ± 0.7 cm over 5 years (with measurements ranging from 16.1 to 21.8 cm). In contrast, the average growth of trees on unused haymaking and pasture lands was lower, at 19.4 ± 0.7 cm, with measurements ranging from 21.1 to 32.3 cm. The second group of samples showed a lesser response to changes in precipitation and temperature.

The correlation analysis results are summarized in Tables 2 and 3. Tree growth was significantly reduced during the hot and relatively dry summer of 2010. In contrast, the summer of 2013, which received substantial rainfall of 408 mm, resulted in the highest average growth increment. Although 2011 had a similar amount of summer rainfall at 322.4 mm, we did not observe any significant growth that year. The precipitation levels during the winters and springs preceding 2011 and 2012 were similar, with totals of 282.7 mm and 240.5 mm, respectively, from October to May, and these figures do not clarify the differences in tree growth. In both 2009 and 2012, despite having comparable precipitation amounts of 312.1 mm and 339.1 mm, we observed notable differences in growth rates. One potential reason for these variations could be the summer temperatures. In years where June, July, and August were cooler, the annual height increment tended to decline (R = − 0.30, though this correlation was not statistically significant), regardless of the rainfall. On the other hand, there was a positive correlation between precipitation and height increase (R = 0.9, p < 0.05). The analysis of the correlation between the amount of precipitation in individual months and the annual growth of young trees allowed us to identify much clearer climatic signals. Trees of different habitats show a synchronous response to the dynamics of local precipitation within a year (Table 2). The most notable synchronization in tree growth was observed in relation to precipitation from the previous October and the current March, where the connection to annual growth increments was statistically significant across all trial plots. Additionally, significant correlations were identified with rainfall in August (96.3% of habitats), May (92.6%), July (81.5%), the previous November (77.7%), and the current June (74.1%). Precipitation in February, December, April, and January showed a statistically significant effect on tree growth in roughly half of the samples, with correlation values ranging from 40.1 to 51.9%. In contrast, rainfall in September had an influence on plant growth in only 18.5% of habitats. Overall, precipitation during 10 of the 12 months examined positively affected the growth of young pine trees, with February and March being the exceptions, as they displayed negative Spearman correlation coefficients.

**Table 2:** The results of testing the statistical significance of Spearman correlation coefficient R between monthly precipitation and annual trees growth in 27 habitats of Scots pine

Months	Cases			
	n.s	P < 0.05	P < 0.01	P < 0.001
Previous year				
October (R = 0.62 ± 0.02)	–	0	–	27
November (R = 0.37 ± 0.03)	6	0	4	17
December (R = 0.16 ± 0.03)	14	8	4	1
Current year				
January (R = − 0.21 ± 0.01)	16	8	3	–
February (R = − 0.27 ± 0.02)	11	5	5	6
March (R = 0.54 ± 0.01)	0	0	0	27
April (R = 0.16 ± 0.03)	14	5	5	3
May (R = 0.54 ± 0.03)	2	2	1	22
June (R = 0.32 ± 0.02)	7	5	1	14
July (R = 0.36 ± 0.03)	5	2	4	16
August (R = 0.43 ± 0.02)	1	1	5	20
September (R = − 0.02 ± 0.03)	22	2	2	1
12 months	98	38	34	154

*n.s.* not significant cases, average R for habitats is shown in parentheses

**Table 3:** The results of testing the statistical significance of Spearman correlation coefficient R between monthly temperature and annual trees growth in 27 habitats of Scots pine

Months	Cases			
	n.s	P < 0.05	P < 0.01	P < 0.001
Previous year				
October (R = 0.17 ± 0.01)	21	5	1	-
November (R = − 0.31 ± 0.02)	5	6	9	7
December (R = − 0.27 ± 0.03)	5	7	10	5
Current year				
January (R = 0.20 ± 0.02)	16	7	2	2
February (R = 0.04 ± 0.02)	27	0	0	0
March (R = − 0.36 ± 0.03)	6	2	1	18
April (R = 0.55 ± 0.02)	0	0	1	26
May (R = − 0.11 ± 0.02)	24	2	1	0
June (R = − 0.18 ± 0.02)	15	9	3	0
J uly (R = 0.13 ± 0.02)	25	1	1	0
August (R = 0.06 ± 0.02)	26	1	0	0
September (R = − 0.59 ± 0.02)	0	0	2	25
12 months	170	40	31	83

*n.s.* not significant cases; average R for habitats is shown in parentheses

The connection between the annual growth increment of young trees and monthly temperatures was clearly demonstrated, as indicated in Table 3. Unlike precipitation, we identified six months that negatively influenced tree growth: the previous November and December, along with the current March, May, June, and September. April and September stood out as the most vital months for growth, showing highly significant Spearman correlation coefficients across all 27 trial plotsIn 81.5% of the samples, significant correlations were noted for the previous November and December. March in 77.8%, January in 59.3%, and June in 44.4%. Additionally, significant correlations between annual growth dynamics and temperature were observed for May, July, and August in several trial plots.

According to the obtained data, abandoned agricultural lands exhibit a higher average annual growth rate of young Scots pines compared to other types of environments. This may be attributed to more favorable growth conditions, such as increased precipitation or improved soil quality. Meadows and pastures show similar average growth rates; however, both environments exhibit lower growth values compared to abandoned agricultural lands. This suggests that these environments may offer less favorable conditions for pine growth (Table 4).

**Table 4:** Average annual growth of young scots pines in three types of habitats

Habitat type	Average growth (cm)	Minimum growth (cm)	Maximum growth (cm)	p-value
Abandoned agricultural lands	24.3 ± 0.7	16.1	32.3	< 0.001
Meadows	19.4 ± 0.7	11.2	21.8	< 0.001
Pastures	19.4 ± 0.7	20.7	38.4	0.567

The p-value is a statistical measure that helps determine whether the obtained results are statistically significant

Table 5 presents the results of calculations for the simplified drought index (DI). The analysis reveals a clear relationship between tree growth and DI values. In 2011, which recorded the highest precipitation (509.9 mm) and relatively low temperature (2.7  °C), the DI reached its maximum value of 428.9, and the annual height increment was the highest, 28.3 cm. This indicates that favorable hydrothermal conditions promote more intensive biomass accumulation. Conversely, in 2012, under high temperatures (4.4 °C) and minimal precipitation (275.1 mm), the DI dropped to 143.1, and tree growth declined to 17.4 cm. This supports the conclusion that drought-related stress significantly reduces growth, even under consistent age and spatial characteristics of the trees.

**Table 5:** Calculations Based on the Simplified Drought Index (DI)

Year	Precipitation (mm)	Temperature (°C)	Drought Index (P − 30 × T)	Pine Growth (cm)
2009	413.5	2.6	413.5 − 78.0 = 335.5	23.7
2010	360.3	3.8	360.3 − 114.0 = 246.3	25.8
2011	509.9	2.7	509.9 − 81.0 = 428.9	28.3
2012	275.1	4.4	275.1 − 132.0 = 143.1	17.4
2013	446.7	2.7	446.7 − 81.0 = 365.7	26.5

In 2010, with a relatively moderate DI (246.3), the growth remained relatively high (25.8 cm), suggesting that elevated temperatures may stimulate growth when moisture availability is adequate. In 2009 and 2013, DI values were 335.5 and 365.7, respectively, which also corresponded to stable growth rates of 23.7 cm and 26.5 cm.

These findings confirm that the simplified drought index effectively reflects the environmental conditions under which annual tree growth occurs and enables the identification of climate tolerance thresholds in young pines. When DI falls below 200, a significant decline in growth can be expected, which is important to consider in the context of climate change and the assessment of the adaptive potential of forest stands.

These results underscore the significance of specific environmental conditions for pine growth and may be used to optimize management strategies to enhance forest productivity across different habitats.

## Discussion

In this study, we examined the annual growth dynamics of 405 young Scots pine trees across 27 trial plots situated in three distinct environmental groups: abandoned arable land, pastures, and grasslands. Despite the relatively homogeneous ecological conditions within these habitats, the trees primarily did not compete with one another for sunlight access. Earlier in Latvia, more than threefold differences in the 5-year growth of young scots pine trees were found in stands with two-fold differences in the initial density (Miezīte et al. 2015). Therefore, we expected that the young trees of our study would fully show the climate signals of only two leading climatic factors, precipitation and temperature, in the growth dynamics. To maximize the detection of this dependence, a 5-year period with alternating contrasting precipitation was chosen. The data obtained confirmed our initial hypothesis. Numerous significant cases of climate response in annual growth were found in almost all months (except February for temperature) in all or many habitats. We found that the amount of precipitation is a more critical ecological factor for the growth of trees in the study region, since a statistically significant correlation between these variables was demonstrated for a larger number of months and samples, and this dependence was significant at higher levels. This may be a region-specific feature of the relatively arid Bashkir Trans-Urals with a strong continental climate. In the northwestern taiga, the role of temperature in Scots pine radial growth was more expressed, and this fact can be explained by the excess of moisture in this region (Lopatin et al. 2008). Similar to our findings, a study by Oberhuber and Gruber (2010) demonstrated a statistical correlation between trees’ radial increments during the growing period from April to August and precipitation values across ecologically diverse habitats. In our analysis of 27 trial plots over a total of 60 months, we observed periods with statistically significant correlations between precipitation and annual tree growth (Table 2), arranged in descending order as follows: previous October, March, August, May, July, previous November, June, February, April, previous December, January, and September. In the first two months listed, the synchronicity of the precipitation response was in all the habitats studied.

The following five months exhibited climate-driven annual tree increments in the majority of trial plots, with percentages ranging from 74.1 to 96.3%. September was identified as the month with the weakest climate signal. Statistically significant correlations between annual tree growth and precipitation levels were observed in only 5 out of the 27 sites studied (18.5%), and four of these sites showed a negative relationship. In the remaining months, a statistically significant correlation was found in about half of the habitats (40.7–59.3%). Except for February, January, and September, we observed only positive significant correlations for all other months. Thus, precipitation might be a climatic factor which have mostly a positive influence on tree increment, starting from autumn preceding the growth of plants until the end of the growing season.

Compared to the effect of precipitation, temperature has less impact on the annual growth of Scots pine in terms of the number of cases of statistically significant correlations and their levels of significance. The second pattern observed was the predominance of a negative correlation between temperature and the trees’ increment. It is possible that a warm spring positively influences the initial growth increment of trees. However, as temperatures rise in the subsequent months, this may hinder growth due to increased evaporation during hot weather, which depletes the essential moisture required by plants (Agafonov and Kukarskikh 2008).

The months of the year were ranked according to their impact on plant growth due to temperature, in descending order as follows: April (positive correlation) and current September (for all 27 trial plots), previous November and December (both the months by 81.5% of trial plots), March (77.8%), June (44.4%), January (40.7%), previous October (22.2%, positive influence), May (11.1%), July (7.4%) and August (3.7%). The absence of any statistically significant effect of temperature on the annual plant increment was observed for February. For every month, significant correlations had either only negative or only positive values in all the trial plots.

The study’s results also indicated that young trees on abandoned agricultural lands in the Uchali region exhibit a higher growth rate compared to other habitats. Since the p-value is less than 0.001, this indicates statistically significant differences between the mean growth rates of young Scots pines across different types of plots. This may be attributed to the more favorable growth conditions on abandoned agricultural lands, such as increased precipitation and improved soil quality.

Overall, the findings emphasize the importance of considering climatic factors in the planning and management of forest resources in the Uchali region. They may also be useful in developing climate adaptation strategies for forestry.

There are not many studies on the relationship between climate and the annual growth of trees in height, and most studies are mainly concerned with the influence of weather features on the formation of tree rings (Kaźmierczak and Zawieja 2014). For critically important months, our results are consistent with the results of other studies on the climate-driven growth of Scots pine.

Shah et al. (2015) explored seasonal climatic signals in Central Siberia by analyzing a long-term tree-ring chronology of *P.* *sylvestris* and reconstructing patterns of annual precipitation. Their findings indicated that the most significant annual precipitation signal occurred over a 12-month period from August to July. The research identified the previous August and November, as well as the current May and June, as the months with the strongest correlations. It was concluded that increased temperatures lead to more pronounced tree growth anomalies, primarily due to insufficient rainfall during July and August. Additionally, the study found a strong link between precipitation from April to June and the growth of *P.* *sylvestris* in a dry valley located in the Eastern Central Alps (Oberhuber and Gruber 2010). For the variety *P.* *sylvestris* var. mongolica, winter precipitation was shown to have a beneficial effect on the growth of young trees. Furthermore, a significant relationship was established between the Palmer drought severity index and tree ring width, spanning from September of the previous year to October of the current year (Liu et al. 2022).

Large-scale forest overgrowing of abandoned agricultural lands in Russia opens up new opportunities for mitigating the effects of climate warming. Agricultural and forestry sectors are attributed as a source of 30% of global emissions leading to climate changes, including 17.4% due to deforestation and forest degradation and 13,5% from agriculture (Jangu and Meena 2011). The “uninvited” appearance of young forests on tens of millions of hectares of Russian unused agricultural land (Schierhorn et al. 2013) has led to the loss of agricultural products. On the other hand, the new forests will mitigate climate change by depositing atmospheric carbon into the biomass (Moomaw et al. 2019) as well as perform such well-known functions as improving soil restoration, circulation of water and organic substances, increasing biodiversity, etc. (Rey Benayas et al. 2007). Forestry management of existing forests is an additional powerful tool for climate change mitigation (Kauppi et al. 2022). Such an opportunity has arisen for new Russian forests that have appeared over the past three decades on abandoned agricultural land due to the adoption of a special decree by the Government in 2021. This regulatory document allowed the multipurpose use of forests on agricultural lands, including plantation cultivation and timber harvesting. Climate warming, which leads to an elongation of the tree growth season, may lead to greater sequestration of atmospheric carbon by boreal forests (Aalto et al. 2022). Our results on clear climate signals in the growth of young Scots pine trees highlight the large adaptability of the species and the increasing potential of stands on abandoned agricultural lands in mitigating climate change.

Thus, the conducted study highlights the significant role of climatic factors, particularly precipitation, in the growth dynamics of young Scots pine in the Bashkir Trans-Urals. The adaptability of Scots pine and the growth potential of plantations on abandoned agricultural lands offer promising avenues for climate change mitigation. The results underscore the necessity of integrating climatic considerations into forest management and planning strategies.

The innovative aspect of the work lies in identifying a clear climatic signal in the growth of young pine trees, specifically the correlation between monthly precipitation and annual growth. The novelty of the research is its examination of the relationship between precipitation and tree growth at a monthly level, providing a more detailed understanding of climatic factors affecting tree growth. Additionally, the study reveals a synchronous response of tree growth to local precipitation dynamics throughout the year, emphasizing the importance of considering local climatic conditions in tree growth research. The study also provides a ranking of months based on their impact on tree growth, which may inform forestry practices and climate adaptation strategies.

The conducted study may attract an international audience due to its global relevance, as climate change and its impact on forest ecosystems are worldwide issues. This makes the research pertinent to scholars, policymakers, and forest managers globally. The detailed analysis and ranking of months may inspire similar studies in other regions, contributing to the development of more accurate climate-forest models. Additionally, the findings could inform sustainable forest management practices, such as optimizing planting and harvesting schedules and creating climate-resilient forest ecosystems. The fusion of ecology, climatology, and forestry has the potential to attract researchers from diverse fields, fostering collaboration and the sharing of insights. Additionally, this research could be replicated in different regions, facilitating worldwide comparisons of climate-forest dynamics and contributing to the formulation of more effective approaches to address climate change.

## Conclusions

The examination of how climatic factors influence the annual growth of young Scots pines across 27 distinct habitats revealed a notable correlation with monthly precipitation and temperature. The results showed that precipitation is crucial for tree growth, especially during the previous October and November, as well as in the current months of March, May, June, and July. The strongest statistically significant relationships between monthly precipitation and annual tree growth were found in October of the previous year (R = 0.62) and March of the current year (R = 0.54), emphasizing the vital role of precipitation during these times.

Temperature also affects the growth of young Scots pines, but its influence tends to be more variable and frequently negative. Specifically, temperature has a particularly adverse effect in the previous November and December, as well as in the current March, May, June, and September. The most significant negative effects were noted in September (R = − 0.59) and March (R = − 0.36), while a positive effect of temperature was observed in April (R = 0.55).

Abandoned arable lands exhibited higher growth rates of young Scots pines compared to unused meadows and pastures. This may be attributed to more favorable soil conditions and greater precipitation on abandoned arable lands.

Overall, the results underscore the importance of considering climatic factors, especially precipitation, in the planning and management of forest resources. The sensitivity of young Scots pine forests to climatic dynamics suggests their potential for atmospheric carbon sequestration, which could have significant ecological implications in the context of climate change.

Understanding the relationship between climatic factors and the growth of young Scots pine trees is critically important for sustainable forest management in Russia. Climate change is expected to have a significant impact on forest ecosystems, and identifying the most crucial months for temperature and precipitation in determining tree growth can aid in informing adaptation strategies to these changes. This research is particularly relevant in the context of the Uchali region, where abandoned agricultural lands are being reforested with Scots pine.

The results obtained can be useful in predicting the impact of climate change on the species. The obtained results may be valuable for developing forest management and land restoration strategies, as they provide insights into the impact of different habitats on tree growth and can be applied to similar regions. In addition, young *P.* *sylvestris* forests on extremely vast abandoned agricultural lands in Russia may play a great role in capturing atmospheric carbon due to their revealed sensitivity to climate dynamics and comparatively fast growth in height. In this regard, the environmental benefits of climate change mitigation may exceed the profits of these lands from agricultural production after their restoration. Therefore, further research is needed to verify this promising opportunity.

## Data Availability

Data will be available on request.

## References

[CR1] Aalto J, Pirinen P, Kauppi PE, Rantanen M, Lussana C, Lyytikäinen-Saarenmaa P, Gregow H (2022) High-resolution analysis of observed thermal growing season variability over northern Europe. Clim Dyn 58(5–6):1477–1493. 10.1007/s00382-021-05970-y

[CR2] Abisheva A, Abishov A, Khairullaeva K, Shynybayev K, Kalissynov B, Maikhin K, Kydyrmanov A, Karamendin K, Valdovska A, Syrym N (2022) AK-2011 strain for the development of a vaccine against equine rhinopneumonitis. Transbound Emerg Dis 69(5):e1972–e1981. 10.1111/tbed.14531 35315978 10.1111/tbed.14531

[CR3] Agafonov LI, Kukarskikh VV (2008) Climate changes in the past century and radial increment of pine in the Southern Ural steppe. Russ J Ecol 39:160–167. 10.1134/S1067413608030028

[CR4] Aguadé D, Poyatos R, Rosas T, Martínez-Vilalta J (2015) Comparative drought responses of *Quercus**ilex* L. and *Pinus**sylvestris* L. in a montane forest undergoing a vegetation shift. Forests 6(8):2505–2529. 10.3390/f6082505

[CR5] Biging GS, Dobbertin M (1995) Evaluation of competition indices in individual tree growth models. For Sci 41(2):360–377. 10.1093/forestscience/41.2.360

[CR6] Bourakba S, Marakhova AI, Stanishevskiy YAM, Vasilenko IA, Zhilkina VY (2024) Exploring the potential of fennel (*Foeniculum**vulgare* Mill.) as a valuable medicinal plant: a comprehensive (review). Drug Dev Registr 13(2):59–67. 10.33380/2305-2066-2024-13-2-1617. (**In Russ.**)

[CR7] Capblancq T, Morin X, Gueguen M, Renaud J, Lobreaux S, Bazin E (2020) Climate-associated genetic variation in *Fagus**sylvatica* and potential responses to climate change in the French Alps. J Evol Biol 33(6):783–796. 10.1111/jeb.13610 32125745 10.1111/jeb.13610

[CR8] Dauylbayev A, Yelmurzayeva R, Kamaljanova T, Ibragimova G (2024) The ambivalence of the implementation of the US arctic policy: integrating and disintegration factors of the allies. Front Polit Sci 6:1341375. 10.3389/fpos.2024.1341375

[CR9] Filipchuk AN, Malysheva NV, Zolina TA, Yugov AN (2020) Boreal forests of Russia: opportunities for climate change mitigation. Forestry 1:92–113. 10.24419/LHI.2304-3083.2020.1.10

[CR10] Fritts HC (1976) Tree rings and climate. Academic Press, Cambridge

[CR11] Golub A, Sohngen B, Cai Y, Kim J, Hertel T (2022) Costs of forest carbon sequestration in the presence of climate change impacts. Environ Res Lett 17(10):104011. 10.1088/1748-9326/ac8ec5

[CR12] Hamrick JL (2004) Response of forest trees to global environmental changes. For Ecol Manag 197(1–3):323–335. 10.1016/j.foreco.2004.05.023

[CR13] Hillam J (1998) Dendrochronology: guidelines on producing and interpreting dendrochronological dates. Ancient Monuments Laboratory, Coveney

[CR14] Jangu S, Meena BL (2011) The role of agriculture in the mitigating global warming. IJDS 5(1):265–282

[CR15] Kamaljanova T, Burakanova G (2020) Natural resource management (NRM) and peacebuilding in case of Liberia. Opcion 91:1–832

[CR16] Kauppi P, Stål G, Arnesson-Ceder L, Hallberg Sramek I, Hoen HF, Svensson A, Wernick IK, Högberg P, Lundmark T, Nordin A (2022) Managing existing forests can mitigate climate change. For Ecol Manag 513:120186. 10.1016/j.foreco.2022.120186

[CR17] Kaźmierczak K, Zawieja B (2014) The influence of weather conditions on annual height increments of Scots pine. Biometr Lett 51(2):143–152. 10.2478/bile-2014-0010

[CR18] Koralewski TE, Wang HH, Grant WE, Byram TD (2015) Plants on the move: assisted migration of forest trees in the face of climate change. For Ecol Manag 344:30–37. 10.1016/j.foreco.2015.02.014

[CR19] Leskinen P, Lindner M, Verkerk PJ, Nabuurs GJ, Van Brusselen J, Kulikova E, Hassegawa M, Lerink B (eds) (2020) Russian forests and climate change. What science can tell us. European Forest Institute, Joensuu. 10.36333/wsctu11

[CR20] Lévesque M, Walthert L, Weber P (2016) Soil nutrients influence growth response of temperate tree species to drought. J Ecol 104(2):377–387. 10.1111/1365-2745.12519

[CR21] Liu Y, Xin Z, Li Z, Maierdang K, Yan T (2022) Response of radial growth of *Pinus**sylvestris* var. mongolica to climate factors in Bashang area of Hebei province. Acta Ecol Sinica 42(5):1830–1840. 10.5846/stxb202010272750

[CR22] Lombardo U (2017) River logjams cause frequent large-scale forest die-off events in southwestern Amazonia. Earth Syst Dyn 8(3):565–575. 10.5194/esd-8-565-2017

[CR23] Lopatin E, Kolström T, Spiecker H (2008) Impact of climate change on radial growth of Siberian spruce and Scots pine in North-western Russia. IFOREST J 1(1):13–21. 10.3832/ifor0447-0010013

[CR24] Machacova K, Bäck J, Vanhatalo A, Halmeenmäki E, Kolari P, Mammarella I, Pumpanen J, Acosta M, Urban O, Pihlatie M (2016) *Pinus**sylvestris* as a missing source of nitrous oxide and methane in boreal forest. Sci Rep 6(1):23410. 10.1038/srep23410 26997421 10.1038/srep23410 PMC4800674

[CR25] Matveev SM (2014) Cycling in dynamics of radial increment in natural and artificial pine stands in central steppe-forest. For Bull 5:110–116

[CR26] Miezīte O, Eglite I, Luguza S, Liepa I (2015) Proceedings of the 7th international scientific conference Rural Development 2015. Height increment of naturally regenerated young forest ftands of Scots pine *Pinus**sylvestris* L. in Myrtillosa forest site type. Aleksandras Stulginskis University, Akademija. 10.15544/RD.2015.076

[CR27] Moomaw WR, Masino SA, Faison EK (2019) Intact forests in the United States: proforestation mitigates climate change and serves the greatest good. Front for Glob Change 2:27. 10.3389/ffgc.2019.00027

[CR28] Nesterenko Y, Nesterenko M, Solomatin N, Khalin A, Vladov Y (2021) Air temperature, precipitation and agronomy in steppe zone of the Southern Urals. IOP Conf Ser Earth Environ Sci 81(1):012076. 10.1088/1755-1315/817/1/012076

[CR29] Oberhuber W, Gruber A (2010) Climatic influences on intra-annual stem radial increment of *Pinus**sylvestris* (L.) exposed to drought. Trees 24:887–898. 10.1007/s00468-010-0458-1 22003269 10.1007/s00468-010-0458-1 PMC3191526

[CR30] Oberhuber W, Kofler W (2000) Topographic influences on radial growth of Scots pine (*Pinus**sylvestris* L.) at small spatial scales. Plant Ecol 146:229–238. 10.1023/A:1009827628125

[CR31] Oikonomakis NG, Ganatsas P (2020) Secondary forest succession in Silver birch (*Betula**pendula* Roth.) and Scots pine (*Pinus**sylvestris* L.) southern limits in Europe, in a site of Natura 2000 network–an ecogeographical approach. For Syst 29(2):81–96. 10.5424/fs/2020292-15680

[CR34] Pan Y, Birdsey RA, Fang J, Houghton R, Kauppi PE, Kurz WA, Phillips OL, Shvidenko A, Lewis SL, Canadell JG, Ciais P, Jackson RB, Pacala S, McGuire AD, Piao S, Rautiainen A, Sitch S, Hayes D (2011) A large and persistent carbon sink in the world’s forests. Science 333(6045):988–993. 10.1126/science.1201609 21764754 10.1126/science.1201609

[CR35] Pecchi M, Marchi M, Giannetti F, Bernetti I, Bindi M, Moriondo M, Maselli F, Fibbi L, Corona P, Travaglini D, Chirici G (2019) Reviewing climatic traits for the main forest tree species in Italy. IFOREST Journal 12(2):173–180. 10.3832/ifor2835-012

[CR36] Pyhäjärvi T, Kujala ST, Savolainen O (2020) 275 years of forestry meets genomics in *Pinus**sylvestris*. Evol Appl 13(1):11–30. 10.1111/eva.12809 31988655 10.1111/eva.12809 PMC6966708

[CR37] Pyörälä J, Liang X, Vastaranta M, Saarinen N, Kankare V, Wang Y, Holopainen M, Hyyppä J (2018) Quantitative assessment of Scots pine (*Pinus**sylvestris* L.) whorl structure in a forest environment using terrestrial laser scanning. IEEE J Sel Top Appl Earth Obs Remote Sens 11(10):3598–3607. 10.1109/JSTARS.2018.2819598

[CR38] Rey Benayas JM, Martins A, Nicolau JM, Schulz JJ (2007) Abandonment of agricultural land: an overview of drivers and consequences. CABI Rev 2007:14–15. 10.1079/PAVSNNR20072057

[CR39] Safonov V (2022) Dependence of antioxidant and biochemical status on selenium content in the blood of animals. Adv Anim Vet Sci 10(2):263–269. 10.17582/journal.aavs/2022/10.2.263.269

[CR40] Safonov V, Ermakov V, Danilova V, Yakimenko V (2021) Relationship between blood superoxide dismutase activity and zinc, copper, glutathione and metallothioneines concentrations in calves. Biomath 10(2):2111247. 10.11145/j.biomath.2021.11.247

[CR41] Schierhorn F, Müller D, Beringer T, Prishchepov AV, Kuemmerle T, Balmann A (2013) Post-Soviet cropland abandonment and carbon sequestration in European Russia, Ukraine, and Belarus. Global Biogeochem Cy 27(4):1175–1185. 10.1002/2013GB004654

[CR42] Seidel H, Schunk C, Matiu M, Menzel A (2016) Diverging drought resistance of scots pine provenances revealed by infrared thermography. Front Plant Sci 7:1247. 10.3389/fpls.2016.01247 27630643 10.3389/fpls.2016.01247 PMC5005371

[CR43] Shah S, Touchan R, Babushkina E, Shishov V, Meko D, Abramenko O, Belokopytova L, Hordo M, Jevsenak J, Kędziora W, Kostyakova T, Moskwa A, Oleksiak Z, Omurova G, Ovchinnikov S, Sadeghpour M, Saikia A, Zsewastynowicz Ł, Sidenko T, Tychkov I (2015) August to July precipitation from tree rings in the forest-steppe zone of Central Siberia (Russia). Tree-Ring Res 71(1):37–44. 10.3959/1536-1098-71.1.37

[CR44] Shiyatov SG, Mazepa VS (2007) Climatogenic dynamics of forest–tundra vegetation at the Polar Urals. Lesovedenie 6:11–22

[CR45] Tchebakova NM, Gerald E, Parfenova E (2006) Impacts of climate change on the distribution of *Larix* spp. and *Pinus**sylvestris* and their climatypes in Siberia. Mitig Adapt Strateg Glob Chang 11(4):861–882. 10.1007/s11027-005-9019-0

[CR46] Venäläinen A, Lehtonen I, Mikko L, Ruosteenoja K, Tikkanen O-P, Viiri H, Ikonen V-P, Peltola H (2020) Climate change induces multiple risks to boreal forests and forestry in Finland: a literature review. Glob Change Biol 26(8):4178–4196. 10.1111/gcb.15183 10.1111/gcb.15183 PMC7383623 32449267

[CR47] Zuidema P, van der Sleen P (2022) Seeing the forest through the trees: how tree-level measurements can help understand forest dynamics. New Phytol 234(5):1544–1546. 10.1111/nph.18144 35478328 10.1111/nph.18144

